# Severe Portal Hypertension in Cirrhosis: Evaluation of Perfusion Parameters with Contrast-Enhanced Ultrasonography

**DOI:** 10.1371/journal.pone.0121601

**Published:** 2015-03-23

**Authors:** Woo Kyoung Jeong, Tae Yeob Kim, Joo Hyun Sohn, Yongsoo Kim, Jinoo Kim

**Affiliations:** 1 Department of Radiology and Center for Imaging Science, Samsung Medical Center, Sungkyunkwan University School of Medicine, Seoul, Korea; 2 Department of Internal Medicine, Hanyang University Guri Hospital, Hanyang University College of Medicine, Guri-si, Gyeonggi-do, Korea; 3 Department of Radiology, Hanyang University Guri Hospital, Hanyang University College of Medicine, Guri-si, Gyeonggi-do, Korea; 4 Department of Radiology, Ajou University Hospital, Ajou University College of Medicine, Suwon-si, Gyeonggi-do, Korea; National Taiwan University Hospital, TAIWAN

## Abstract

**Objective:**

To investigate the role of contrast-enhanced ultrasonography (CEUS) and Doppler ultrasonography (DUS) in the diagnosis of severe portal hypertension (PH) in patients with liver cirrhosis (LC).

**Methods:**

Patients with PH scheduled to receive hepatic venous pressure gradient (HVPG) measurement were recruited for this study. Hepatic DUS and CEUS were performed successively. Several Doppler and CEUS parameters were explored for correlation with HVPG values and their association with severe PH (≥ 12 mmHg of HVPG). Comparison of the parameters between the severe and non-severe PH groups and their correlation with HVPG values was evaluated. A receiver operating characteristic (ROC) curve analysis was also performed to investigate the performance in order to diagnose severe PH.

**Results:**

Fifty-three consecutive patients were enrolled in this study. Among them, 43 patients did not have significant ascites. Compared with the non-severe PH group, portal venous velocity and intrahepatic transit time (ITT) were significantly reduced in the severe PH group (all p<0.05). Difference between inspiratory and expiratory hepatic venous damping indices (ΔHVDI), hepatic venous arrival time (HVAT) and ITT moderately correlated with HVPG (*r* = -0.358, -0.338, and -0.613, respectively). Areas under the curves for severe PH were 0.94 of ITT and 0.72 of HVAT, respectively (all p<0.05). ITT under 6 seconds indicated severe PH with a sensitivity of 92% and a specificity of 89%.

**Conclusions:**

Hepatic CEUS may be more useful in estimating the HVPG value and determining the presence of severe PH compared to DUS, and ITT was the most accurate parameter to diagnose severe PH.

## Introduction

Currently, contrast-enhanced ultrasonography (CEUS) is emerging as a novel modality for liver imaging [1, 2]. Not only is it applied for the detection and diagnosis of focal hepatic lesions such as hepatocellular carcinoma and metastasis [3–5], it is also used for quantitative evaluation of treatment response after chemotherapy for malignant tumors [1, 2].

Some investigators have expanded the indication of CEUS to the evaluation of diffuse liver disease. Hepatic vein arrival time (HVAT) is known to reflect the severity of hepatic fibrosis [6, 7]. In a recent study, CEUS was demonstrated to be a feasible modality in assessing the severity of portal hypertension (PH) [8, 9] with the potential of substituting hepatic venous pressure gradient (HVPG). HVAT was shortened in patients with clinically significant PH (≥ 10 mmHg of HVPG) because of the development of intrahepatic shunts from the hepatic artery to the hepatic vein in cirrhotic patients with high portal venous pressure [8].

Although HVAT measurement is simple, it is not sufficient to reflect changes in the perfusion status of cirrhotic livers. For example, it is possible that HVAT is vulnerable to the administration of contrast agent, difference of circulatory function [10], and the presence of extrahepatic shunts [11]. To overcome these limitations, the intrahepatic transit time from arterial arrival to venous arrival was considered as a new and improved parameter in several investigations [10, 12, 13]. Furthermore, some investigations have tried to explain that perfusion parameters derived from the time-intensity curve of CEUS, such as peak signal intensity (PSI) or time to peak (TTP), might be useful as complementary tools in the evaluation of liver fibrosis [14, 15]. In comparison, no study has assessed whether CEUS parameters have a role in estimating the grade of PH.

Along with CEUS, hepatic Doppler ultrasonography (US) is a non-invasive method to investigate the hemodynamic status of the liver in various conditions including evaluation of the severity of PH in chronic liver disease patients [16–19]. In particular, dampening of the flow wave on hepatic venous Doppler US was an important and well-known sign of high portal pressure. Damping index not only reflects portal pressure but also liver dysfunction [18]. Portal venous velocity is also an important parameter that decreases in severe PH [16]. However, there is a lack of comparative study between CEUS and Doppler parameters as far as we know.

The purpose of this study is to investigate the perfusion parameters in CEUS and to compare them with those of Doppler US examination in determining the severity of PH in patients with liver cirrhosis.

## Materials and Methods

### Ethics statement

This cross sectional study was approved by the institutional review board of Hanyang University Guri Hospital in April 2011. The research was conducted from May 2011 to February 2012. Written informed consent was obtained from all subjects.

### Subject

**Fig 1:**
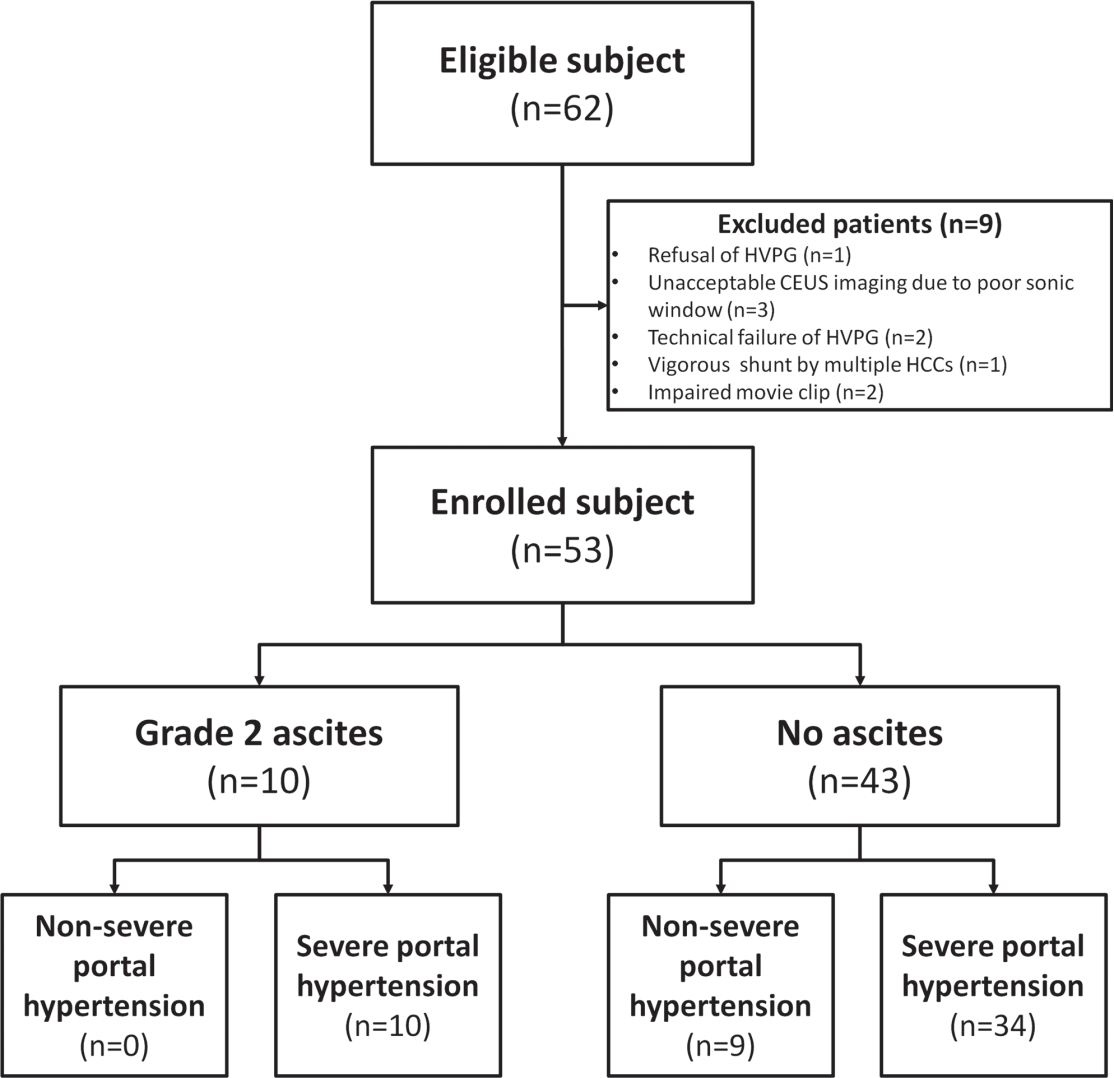
Diagram of study design and recruitment of the patients.

We prospectively recruited consecutive patients with portal hypertension who were scheduled for HVPG measurement and agreed to engage in this study (Fig. 1). HVPG measurement was performed in patients with previous history of variceal bleeding or impending sign of variceal bleeding in order to evaluate the presence of PH and assess the clinical course of PH. The inclusion criteria for the subjects were as follows: (1) age ranging from 20 to 70 years; (2) clinically or histologically confirmed liver cirrhosis; (3) consent to HVPG measurement for this study; and (4) a Child-Pugh score of less than twelve. Exclusion criteria were as follows: (1) baseline systolic arterial blood pressure of < 90 mmHg; (2) intractable ascites or obvious hepatic encephalopathy; (3) malignant hepatic tumour including HCC; (4) cerebrovascular disease, sepsis and uremia; (5) severe jaundice (total bilirubin level of > 10 mg/dL); (6) serum creatinine level of > 1.5 mg/dL; (7) pregnant women; (8) contraindication to CEUS study including severe left-to-right shunt.

### Doppler and CEUS Perfusion US

For Doppler and CEUS study, an ultrasound machine (iU22, Philips Medical System, Bothell, WA) with a 5–1-MHz convex-array transducer was used. All CEUS and Doppler examinations were performed by the same radiologist (W.K.J.) with 8-years of experience in performing liver US as part of his regular practice. Before the examination, the patients were prepared on the fasting status for longer than 8 hours. First, a hepatic Doppler examination was performed. Hepatic venous Doppler waveforms were obtained from the right or middle hepatic vein within 3-cm distance from the confluence with the IVC. Taking into account the possibility that changing intra-abdominal pressure by breathing could influence venous flow in the IVC and hepatic vein, Doppler waveform was obtained during both expiration and inspiration without suspending respiration. The detailed method of the Doppler examination was described in our previous work [17]. Portal venous velocity (PVV) was also measured in the main portal vein. All Doppler angles were kept at less than 60° and Doppler waveforms and PVV were recorded in triplicate. In addition, the maximum and minimum velocity of the retrograde systolic wave was measured for calculating hepatic venous damping indices (HVDI) as Doppler parameters: the ratio of the minimum to the maximum velocity of the hepatic venous flow during systolic period [18]. HVDIs were classified into two indices according to the different respiratory phases: inspiratory HVDI (HVDI_*i*_) and expiratory HVDI (HVDI_*e*_). Additionally, the difference of respective HVDI (ΔHVDI) was also calculated [17].

After the Doppler US exam, the examiner held the transducer in a fixed position in order to acquire an intercostal scan of the right liver. The scanning parameters of CEUS were as follows: maximum depth 11–14 cm; mechanical index, 0.06; frame rate, 8–10 Hz; fixed overall gain, 88%; dynamic range, 40 dB; general contrast mode. The patients were asked to breathe regularly and gently throughout the CEUS examination. Thereafter, a 2.4 mL bolus of microbubble contrast agent (sulphur hexafluoride, Sonovue, Bracco SPA, Milan, Italy) was injected manually into the left antecubital vein through a 20-guage needle at a rate of 1 mL/s, followed by 5-mL of normal saline for flushing which was delivered at the same rate. CEUS imaging was recorded from 10 seconds before contrast injection in order to observe the baseline signal intensity of the hepatic vasculature and parenchyma, and maintained for up to 3 minutes. A video clip was recorded for subsequent analysis of the time-intensity curve for hepatic perfusion.

### HVPG measurement

The patients were taken off any medication that could have potentially affected their splanchnic hemodynamic status, such as non-selective beta blockers, at least a week prior to HVPG measurement. HVPG measurement was performed by one radiologist (J.K.) with experience in more than 50 HVPG measurements. He was blinded to the results of the DUS and CEUS study. For hepatic vein catheterization, a sheath introducer was inserted into the right internal jugular vein under local anaesthesia. Vital signs were monitored during the procedure which included electrocardiography, blood pressure, and pulse oximetry. After catheterization of the hepatic vein, a 6-Fr balloon tip catheter (Arrow International, Inc., Cleveland, OH) was advanced under fluoroscopic control into the right hepatic vein. Before the pressure measurements, a hepatic venogram was obtained to check that the hepatic vein was completely wedged by the inflated balloon and that there was no shunt between the occluded hepatic vein and other hepatic veins. The balloon was then deflated and the catheter flushed with normal saline.

Free hepatic vein pressure was measured less than 5 cm away from its confleunce into the inferior vena cava, using an electronic barometer built into the patient monitoring equipment (Pulscan-combo, Scionic Co. Ltd, Korea). Wedged hepatic vein pressure was measured at the same location while the balloon was inflated. All the measurements were performed in triplicate, and the results were reported as the means of the three measurements. The HVPG was determined by subtracting the free hepatic venous pressure from the wedged hepatic venous pressure. After measuring HVPG, the pressure in the inferior vena cava around the hepatic vein was also measured for comparison with the free hepatic vein pressure. According to the previous consensus concerning the clinical significance of HVPG, the subjects were classified using a HVPG threshold: severe PH was defined as HVPG of ≥ 12 mmHg, which is predictive of variceal bleeding [20].

### Analysis of the CEUS perfusion data

After collecting the data from all the subjects, two readers were asked to perform quantitative analyses of the CEUS perfusion study: a board-certified radiologist (W.K.J.) and hepatologist (T.Y.K.) with eight and nine years of clinical experience, respectively. The reviewing process of perfusion imaging was performed independently, without any clinical data. To avoid recall bias, all data were evaluated within at least a 4-weeks from the time of acquisition.

**Fig 2:**
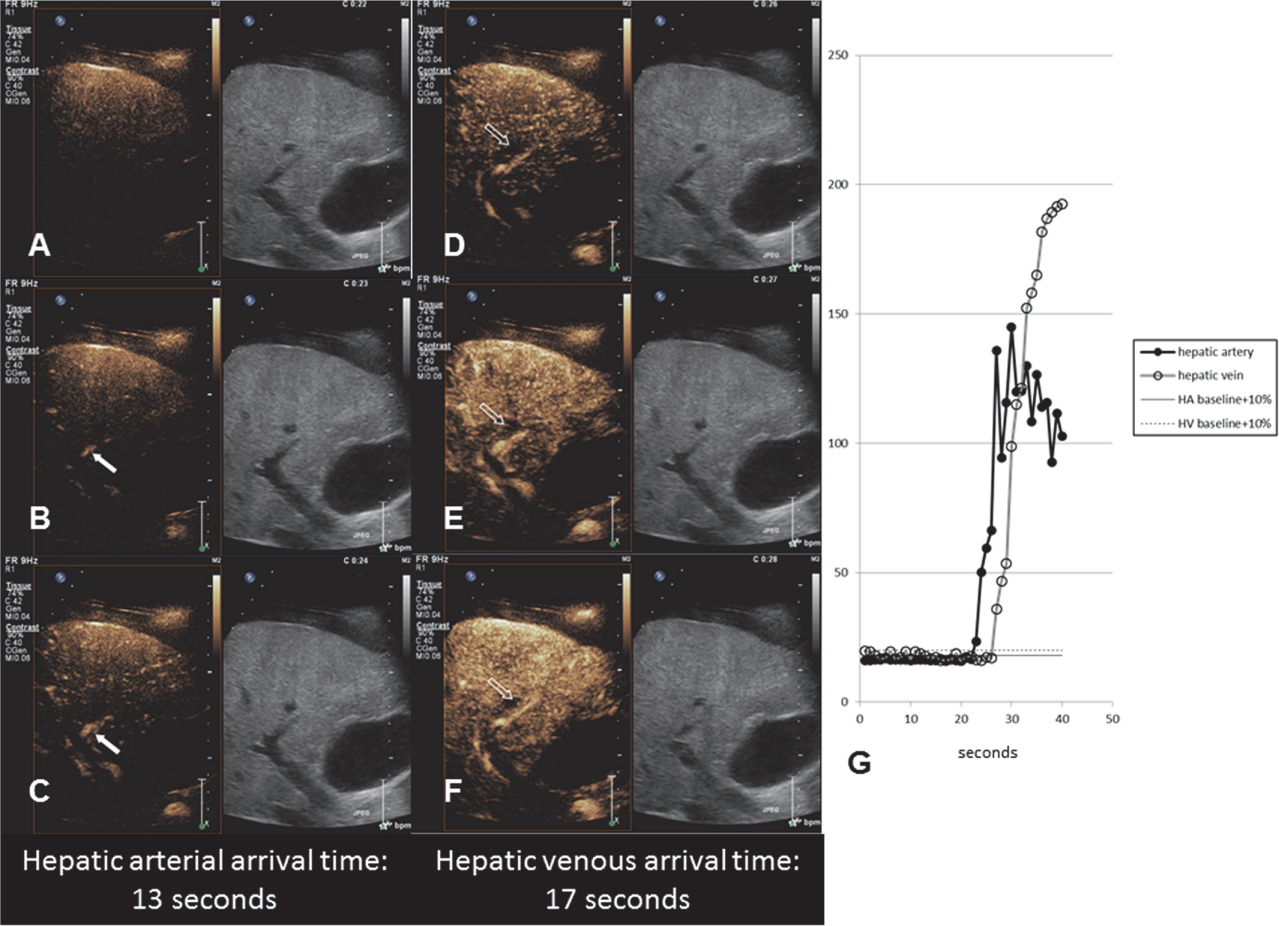
Measurement of vascular arrival time. Serial contrast enhanced ultrasonograms of a 49-year old man with Child C liver cirrhosis show microbubbles appearing in the hepatic artery (arrows in A, B, C) at 23 seconds (13 seconds after contrast injection) and hepatic vein (open arrows in D, E, F) at 27 seconds. After drawing the ROI, the acoustic intensity of the region of interest in the vessels is tracked stack images and measured per second. The arrival times is defined as the time to reach to 10% above the baseline intensity.(G) Hepatic arterial and venous arrival time is 13 and 17 seconds, respectively, resulting in an intrahepatic transit time of 4 seconds.

For quantitative analysis of the CEUS perfusion data, an open-source image analysis program (ImageJ software, National Institutes of Health, Bethesda, MD) was used. Saved video clips were converted into motion JPEG and loaded with ‘virtual stack’ mode on the ImageJ software. Analyses of CEUS perfusion study consisted of measurement of the time-to-arrive in hepatic vessels and analysis of the time-intensity curve of the hepatic parenchyma. For measurement of the arrival time, the readers drew regions of interest (ROI) on the portal triad at the hepatic hilus and right hepatic vein. They then tracked stack images to check and adjust the ROI within the vascular structures, and measured the acoustic intensity (arbitrary unit, A.U.) in the ROI per second. (Fig. 2) The obtained data were inputted into a Microsoft Excel spreadsheet, and a time-intensity curve was plotted. The mean intensity values measured at the period before contrast injection (the first ten seconds) were regarded as the baseline intensity. Arrival time was defined as the time to reach 10% above the baseline intensity [13]. The readers observed two arrival times: hepatic artery and hepatic vein arrival times (HAAT and HVAT). Intrahepatic transit time (ITT), which was defined as the difference between HVAT and HAAT, was also calculated [10].

**Fig 3:**
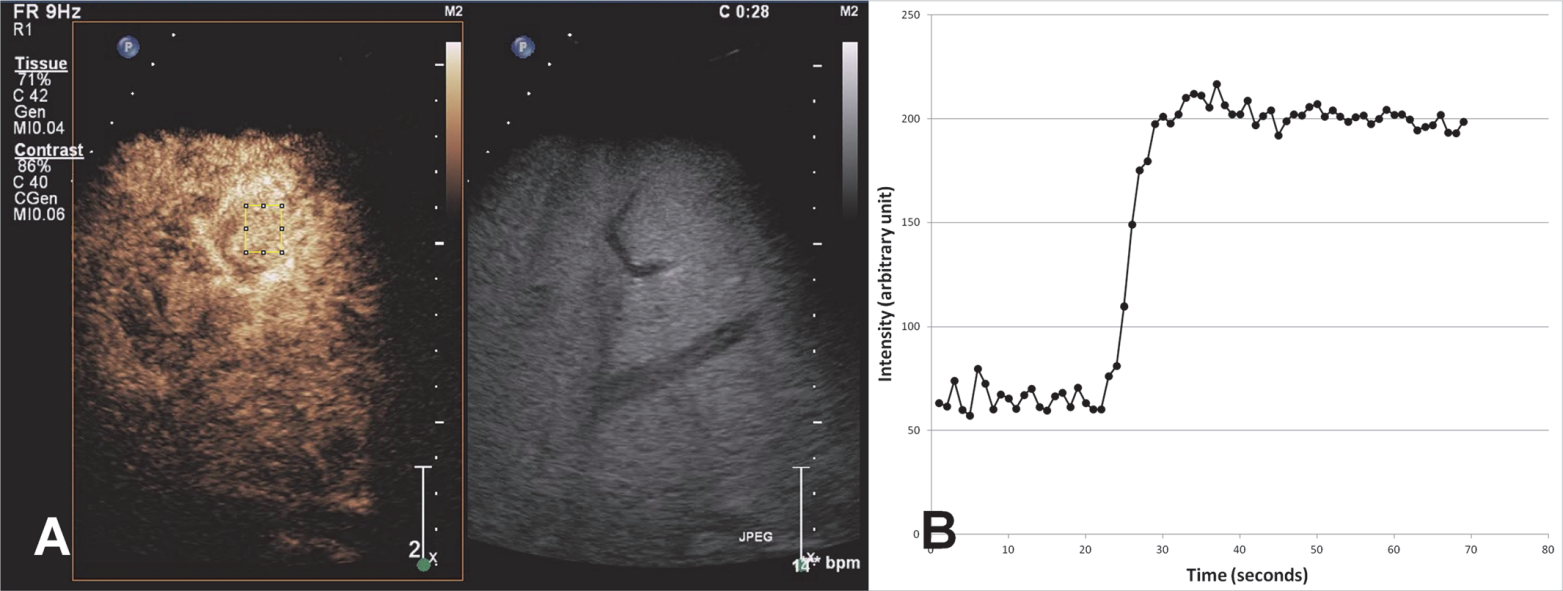
Peak signal intensity (PSI) and time to peak (TTP). A contrast enhanced ultrasonogram of a 53-year old man with Child C liver cirrhosis shows a region of interest (ROI) located on the hepatic parenchyma.(A) Two parameters are obtained from the time-intensity curve. PSI is defined as the difference between the maximum acoustic intensity and baseline, and TTP is defined as the time to reach PSI from the time of injection.(B).

Next, the readers drew another ROI on the liver parenchyma taking care to avoid any major vessels. The parenchymal ROI was located about 3 cm away from the hepatic capsule. Its depth ranged from 5 cm to 10 cm under the transducer. Then, they obtained a time-intensity curve in the manner written above and explored two perfusion parameters: PSI and TTP of the curve (Fig. 3) [15]. PSI was defined as the difference between the maximum acoustic intensity and baseline intensity, and TTP was defined as the time to reach PSI from injection. Among these parameters, HVAT, ITT, PSI and TTP were considered as eligible parameters which were related to the HVPG and severity of PH.

### Statistics

All laboratory data and parametric data derived from Doppler and perfusion studies were presented as the mean and standard deviation after verifying normal distributions with Kolmogorov-Smirnov test. The mean values of each parameter were compared between non-severe PH and severe PH group using Student t-test. To evaluate the interobserver agreement level of CEUS parameters, intraclass correlation coefficient (ICC) between two measurements by reader 1 and 2 were calculated. To assess the correlation with HVPG, Pearson’s correlation test was performed. In addition, we also investigated the presence of grade 2 ascites evident by moderate symmetrical distension of abdomen because increased intraabdominal pressure by ascites could influence splanchnic circulation [21]. Then we performed the assessment of the diagnostic performance to the patients without moderate ascites.

To investigate and compare the diagnostic performance of severe PH in the patients without moderate ascites, a receiver operating characteristic (ROC) curve analysis was also performed. Using these ROC curves, we also calculated the sensitivity, specificity, accuracy, positive and negative predictive values when the optimal cut-offs of the parameters were applied. The differences among the AUROCs were assessed using DeLong’s test and the comparison of the sensitivity, specificity and accuracy of the parameters was performed using McNemar’s test. Bonferroni correction was also applied for comparison of four parameters (PVV, ΔHVDI, HVAT and ITT). In addition, the proportion of misdiagnosed patients (out of the cut-off value) was also calculated. Statistical analyses were performed using MedCalc for Windows (version 12.7.0.0; MedCalc Software, Mariakerke, Belgium). A *p* value of <0.05 was considered significant.

## Results

### Enrolled subjects

For this study, 62 eligible subjects underwent CEUS imaging. Among these subjects, 9 patients were excluded. Consequently, 44 consecutive patients were respectively enrolled in the severe PH groups and the remaining 9 patients were enrolled in the non-severe PH group. Among the subjects, there were 43 patients without significant ascites and 34 patients (79%) who did not have grade 2 ascites were classified to severe PH. Alcoholic consumption (n = 32; 60%) was the most common cause of liver diseases, followed by hepatitis B viral infection (n = 13; 25%). There were 25 (47%) patients in the Child-Pugh A category, 23 (43%) in Child-Pugh B, and 5 (9%) in Child-Pugh C. All laboratory data were normally distributed and platelet count and prothrombin time were significantly different between severe and non-severe groups with regards to the laboratory features (Table 1).

**Table 1:** Demographic data of the subjects.

Subjects		Non- severe (n = 9)	Severe (n = 44)	P-value*
Age, year		54.1±9.1^†^	55.1±9.0	0.763
Male gender, numbers (%)		8 (89%)	27 (61%)	0.112
Underlying disease	Alcoholic	7	25	0.531
	HBV infection	2	11	
	HCV infection	0	4	
	Others	0	4	
Laboratory findings	Bilirubin (mg/dL)	0.93±0.48	2.31±3.65	0.267
	Albumin (mg/dL)	3.44±1.00	3.03±0.63	0.110
	Creatinine (mg/dL)	0.90±0.30	0.83±0.21	0.382
	AST (IU/L)	50.2±26.9	66.8±66.2	0.467
	ALT (IU/L)	33.2±13.2	27.8±32.0	0.623
	Platelet (/mm^3^)	151.1K±60.5K	85.6K±45.7K	0.001
	PT (INR)	0.98±0.13	1.14±0.20	0.027
Decompensated LC (%)		7 (78%)	36 (82%)	0.778
Moderate ascites (%)		0 (0%)	10 (23%)	0.180
Child-Pugh score		6 (5–9)	7 (3–12)	0.267
Child-Pugh class (A/B/C)		6/3/0	19/20/5	0.341
MELD score		7 (6–12)	9 (6–20)	0.304
Mean HVPG (mmHg)		8.2±3.4	17.1±3.7	<0.001

*P-values were calculated by independent Student’s t-tests, chi-square test, or Mann-Whitney test. All laboratory data were normally distributed.

^†^Numbers are mean ± standard deviation

PH = portal hypertension; HBV = hepatitis B virus; HCV = hepatitis C virus; AST = aspartate aminotransferase; ALT = alanine aminotransferase; PT = prothrombin time; MELD = model for end-stage liver disease; HVPG = hepatic venous pressure gradient.

### Doppler and CEUS perfusion parameters

The mean values of Doppler and CEUS perfusion parameters, which are summarized in Table 2, were within normal distribution. One patient could not undergo Doppler study because of poor cooperation. Among the Doppler parameters, HVDI_*e*_ was in the non-severe and severe PH groups. In contrast, HVDI_*i*_, ΔHVDI, and PVV tended to decrease in severe PH. However, only the mean PVV was significantly different between the severe and non-severe PH groups (p = 0.007). Regarding the CEUS perfusion parameters, the ITT of the PH group was significantly decreased (p<0.001 by both readers). HVAT was also decreased on the result of reader 1 (p = 0.045) and pooled data (p = 0.005).

**Table 2:** Mean Doppler and CEUS perfusion parameters in the subjects.

Parameters			< 12 mmHg (n = 9)	≥ 12 mmHg (n = 44)	P-value*
Doppler parameters	HVDI_*e*_		0.55±0.17	0.57±0.16	0.774
	HVDI_*i*_		0.74±0.21	0.66±0.19	0.269
	ΔHVDI		0.19±0.16	0.09±0.14	0.073
	PVV		20.4±5.9	15.9±3.9	0.007
CEUS perfusion parameters	Reader 1	HVAT	23.0±3.2	19.8±4.4	0.045
		ITT	7.9±1.3	4.9±1.5	<0.001
		PSI	117.2±29.9	110.1±21.4	0.400
		TTP	52.9±9.7	48.1±14.6	0.353
	Reader 2	HVAT	22.9±3.0	19.8±4.6	0.061
		ITT	8.0±1.4	5.3±1.6	<0.001
		PSI	115.7±28.5	110.4±24.2	0.564
		TTP	40.8±9.4	32.6±13.0	0.082
	Pooled data	HVAT	22.9±3.0	19.8±4.4	0.005
		ITT	7.9±1.3	5.1±1.5	<0.001
		PSI	116.5±28.4	110.3±22.7	0.314
		TTP	46.8±11.1	40.4±15.8	0.102

*P-values were calculated by independent Student’s t-tests.

PH = portal hypertension; HVDI_*e*_ = expiratory hepatic venous damping index; HVDI_*i*_ = inspiratory hepatic venous damping index; ΔHVDI = difference between inspiratory and expiratory hepatic venous damping indices; PVV = portal venous velocity; HVAT = hepatic venous arrival time; ITT = intrahepatic transit time; PSI = peak signal intensity; TTP = time to peak

Looking into the correlation with HVPG, ΔHVDI moderately correlated with HVPG (*r* = -0.358; p = 0.009), but PVV did not significantly correlate with HVPG (*r* = -0.266; p = 0.055). In the CEUS perfusion parameter, HVAT and ITT were better correlated with HVPG than any Doppler parameters. The correlation coefficients of HVAT were -0.341 by reader 1 and -0.336 by reader 2. ITT was the best correlated parameter to estimate HVPG (*r* = -0.568 by reader 1 and -0.628 by reader 2). In the patients without significant ascites, correlation coefficients of PVV and ΔHVDI were slightly decreased, but those of HVAT and ITT were reversely increased. Additionally, TTP by reader 2 was also significantly correlated with HVPG (*r* = -0.308; P = 0.025). However, PSI by both readers and TTP by reader 1 did not correlate significantly with HVPG (Table 3).

**Table 3:** Correlation coefficients between the parameters and HVPG.

Parameters			Correlation coefficients (*r*) *	
			All subject	patients without significant ascites
Doppler parameters	HVDI_*e*_		0.083	0.030
	HVDI_*i*_		−0.203	−0.222
	ΔHVDI		−0.358	−0.334
	PVV		−0.260	−0.203
CEUS perfusion parameters	Reader 1	HVAT	−0.341	−0.405
		ITT	−0.568	−0.607
		PSI	−0.061	−0.116
		TTP	−0.209	−0.158
	Reader 2	HVAT	−0.336	−0.372
		ITT	−0.628	−0.623
		PSI	−0.067	−0.125
		TTP	−0.308	−0.275
	Pooled data	HVAT	−0.338	−0.388
		ITT	−0.597	−0.613
		PSI	−0.064	−0.120
		TTP	−0.223	−0.190

*Pearson’s correlation coefficients

PH = portal hypertension; HVDI_*e*_ = expiratory hepatic venous damping index; HVDI_*i*_ = inspiratory hepatic venous damping index; ΔHVDI = difference between inspiratory and expiratory hepatic venous damping indices; PVV = portal venous velocity; HVAT = hepatic venous arrival time; ITT = intrahepatic transit time; PSI = peak signal intensity; TTP = time to peak

Between reader 1 and 2, the ICC for the interpretation of HVAT, ITT, and PSI were 0.938 (95% confidence interval, 0.894 to 0.964), 0.860 (0.769 to 0.917) and 0.872 (0.788 to 0.924), respectively. However, the correlation coefficient of TTP was only moderate (ICC = 0.394).

### Performance for diagnosis of severe portal hypertension

**Fig 4:**
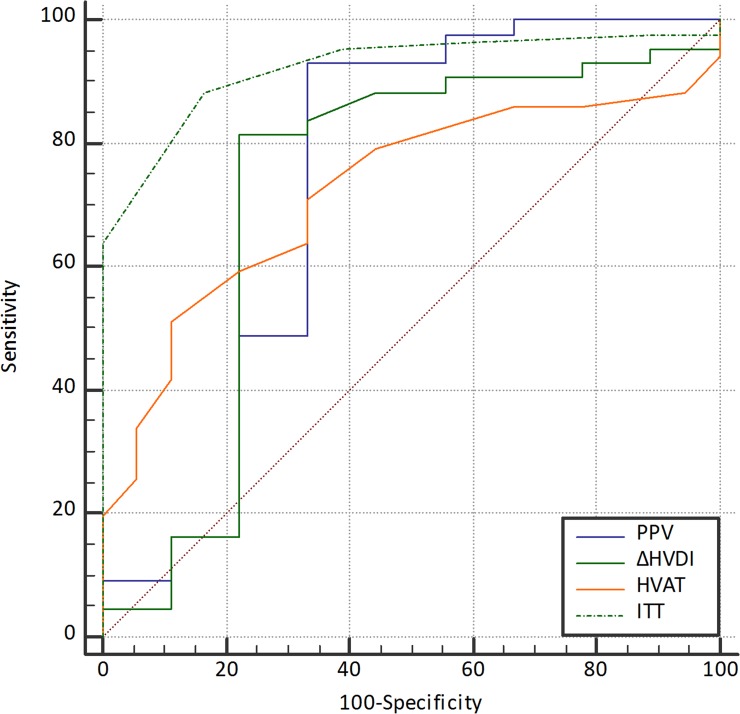
Receiver operating characteristic curves of Doppler and perfusion parameters for estimating severe portal hypertension. The area under the curve (AUC) of intrahepatic transit time (ITT) is larger than any others (0.924), and the second and third largest AUCs are portal venous velocity (PVV; 0.731) and hepatic venous arrival time (HVAT; 0.726), respectively.

The areas under the ROC curves of PVV, ΔHVDI and HVATfor the prediction of severe PH were similar to one another. (Table 4 and Fig. 4) However, the area under the ROC curve (AUROC) of ITT was larger than others and significantly higher than that of HVAT. The other CEUS perfusion parameters including PSI, and TTP did not help to determine the presence of severe PH (AUROC = 0.62 and 0.60, respectively; p-values are larger than 0.05). The optimal cut-off was ITT of 6 seconds with a sensitivity of 85.3% to 91.2% and specificity of 77.8% to 88.9%. In the case of HVAT, a cuf-off of 19 seconds or less had a sensitivity of 88.9% sensitivity and specificity of 58.1% to 62.8% for diagnosing severe PH (Table 4). False positive rate of all parameters were not relatively high, and false negative rate of ITT was lower than that of HVAT (Table 5).

**Table 4:** AUROC of parameters and diagnostic performance after applying optimal cut-off levels of parameters for the diagnosis of severe portal hypertension.

	PVV	ΔHVDI	HVAT		ITT		P-values*		
			Reader 1	Reader 2	Reader 1	Reader 2	ITT vs. PVV	ITT vs. ΔHVDI	ITT vs. HVAT
AUROC	0.71 (0.55–0.84)	0.69 (0.53–0.83)	0.72 (0.57–0.85)	0.71 (0.55–0.84)	0.94 (0.83–0.99)	0.90 (0.77–0.97)	0.125	0.072	<0.001
Cut-off	≤20.8 cm/s	≤0.18	≤ 19 sec		≤ 6 sec		―	―	―
Sensitivity (%)	91.2	79.4	55.9	50.0	91.2	85.3	>0.999	0.344	0.002
Specificity (%)	66.7	77.8	88.9	88.9	88.9	77.8	0.625	>0.999	>0.999
Accuracy (%)	86	79.1	62.8	58.1	90.7	83.7	0.754	0.266	0.004
PPV (%)	91.2	93.1	95.0	94.4	96.9	93.5	―	―	―
NPV (%)	66.7	50.0	34.8	32.0	72.7	58.3	―	―	―

HVAT = hepatic venous arrival time; ITT = intrahepatic transit time; ΔHVDI = difference between inspiratory and expiratory hepatic venous damping indices; PVV = portal venous velocity; PPV = positive predictive value; NPV = negative predictive value

* P-values were calculated by McNemar tests among 4 parameters (PVV, ΔHVDI, HVAT and ITT by reviewer 1) and <0.0083 of p-value was considered as significant difference between two parameters (Bonferroni’s correction).

**Table 5:** Proportion of misdiagnosed patients when the optimal cut-offs were applied.

	PVV	ΔHVDI	HVAT		ITT	
			Reviewer 1	Reviewer 2	Reviewer 1	Reviewer 2
True positive	31 (72%)	27 (63%)	19 (44%)	17 (40%)	31 (72%)	29 (67%)
True negative	6 (14%)	7 (16%)	8 (19%)	8 (19%)	8 (19%)	7 (16%)
False positive	3 (7%)	2 (5%)	1 (2%)	1 (2%)	1 (2%)	2 (5%)
False negative	3 (7%)	7 (16%)	15 (35%)	17 (40%)	3 (7%)	5 (12%)

HVAT = hepatic venous arrival time; ITT = intrahepatic transit time; ΔHVDI = difference between inspiratory and expiratory hepatic venous damping indices; PVV = portal venous velocity

## Discussions

This study showed that CEUS perfusion imaging is able to determine the severity of PH. In addition, ITT, which is defined as the time delay from the arrival of microbubbles at the hepatic artery to the hepatic vein, was the most valuable perfusion parameter for this purpose. The concept of ITT was developed from the study of Sugimoto [22]. They found that ITT allowed for correction of differences in the cardiopulmonary transit. In particular, ITT may be more useful than HVAT in cirrhotic patients because they are vulnerable to hepatopulmonary syndrome, which is characterized by a defect in arterial oxygenation induced by pulmonary vascular dilatation or intrapulmonary shunt [23]. The prevalence of hepatopulmonary syndrome has been reported to range from 4% to 19% in cirrhotic patients [24]. As with the theory of HVAT [8], intrahepatic arteriovenous shunt and arterialization of capillary beds formed by intrahepatic hemodynamic change could account for earlier ITT seen in patients with cirrhosis [7, 25, 26]. ITT decreased with the progression of fibrosis, a finding that is consistent with some reports [12, 13, 22, 27]. However, its relationship with the severity of portal hypertension has not yet been revealed. Indeed, there is not much literature on CEUS analysis for grading of portal hypertension [8, 9].

Although the diagnostic performance of HVAT for clinically significant PH in the study by Kim and his colleagues (areas under the curves = 0.973 vs. 0.980) was similar to our result, the correlation between HVAT and HVPG was better (R^2^ = 0.55 in compensated liver cirrhosis (LC) and 0.46 in decompensated LC). The first possible cause is that the patients with decompensated LC comprised the majority of the population in this study. In decompensated LC, the increased portal blood flow may be influenced not only by intrahepatic shunt but also by systemic hemodynamic status including cardiac output. It is possible that HVAT in decompensated LC does not reflect intrahepatic hemodynamic changes as much as that in compensated LC [8]. Nevertheless, it is possible that the measurement of arrival time, which is a simple and reproducible method, could be utilized for determining the severity of PH as well as grading hepatic fibrosis. Unfortunately, other CEUS parameters, such as PSI and TTP, did not seem to play a role in determining the severity of PH in this study. However, PSI tended to decrease in patients with severe PH. In a previous study, these parameters were investigated for predicting liver fibrosis in chronic hepatitis C patients [15], and demonstrated that the PSI of the liver parenchyma inversely correlated well with the fibrosis scores by fast pass of microbubbles in a decreased sinusoidal volume [28]. TTP was weakly correlated with HVPG, but the main problem of TTP was that the interobserver agreement was very low. This was because there was a broad, plateau-like peak on the time-intensity curve of the liver parenchyma (Fig. 3), and because the decision on the location of the peak could be dispersed according to the readers, which contrasts with the PSIs.

Along with CEUS, hepatic Doppler US has been widely utilized for estimation of the severity of diffuse liver diseases including PH [17–19, 29–31]. Doppler US is a method that can be used to observe hemodynamic changes in patients with PH and some Doppler parameters have been introduced [17, 18]. Also in this study, the Doppler parameters derived from portal and hepatic venous flow pattern were significantly related to HVPG and the diagnosis of severe PH. However, there are some drawbacks in Doppler-derived parameters for determining the severity of diffuse liver diseases. First, ultrasonography including Doppler US depends on the level of the operator’s skill, thus making the Doppler parameters difficult to reproduce with reliability [26]. Although nothing has been proven about the reproducibility of CEUS perfusion imaging yet, this study showed that the interobserver variability with regards to the interpretation of arrival time was acceptable.

Thus, CEUS may be an alternative method to overcome these problems related to US evaluation of PH. However, it is necessary to set up a standardized protocol for CEUS perfusion imaging in order to improve the reliability of the study.

There are some limitations to this study. First, the number of enrolled patients in both the control and disease groups was relatively small. Second, the study population was somewhat heterogeneous and skewed to the group with severe disease. However, measurement of HVPG was generally considered for patients with a high risk of severe complications of PH. Therefore, it may be reasonable that CEUS and Doppler examination should be performed in order to predict severe complications of PH. Third, we could not help obtaining semi real-time perfusion data for each second because the oscillation of signal intensity and respiration artifact could interfere to the data acquisition. Finally, the interobserver variability of the Doppler study was not evaluated because Doppler examination was performed as a routine examination for patients with portal hypertension.

In conclusion, Hepatic CEUS may be useful for estimating the severity of PH, along with Doppler US. ITT was the most accurate parameter to diagnose severe PH. If the proper prospective studies are conducted in the future to overcome the limitations of this study, there is a potential that CEUS perfusion imaging may become widely accepted as a substitute for invasive methods of measurement such as HVPG measurement, especially in cirrhotic patients who face the risk of variceal bleeding.

## Supporting Information

S1 Dataset All laboratory, Doppler, CEUS parametric data of enrolled subject. (XLSX)

## References

[1] Zhou JH, Cao LH, Liu JB, Zheng W, Liu M, Luo RZ, et al Quantitative assessment of tumor blood flow in mice after treatment with different doses of an antiangiogenic agent with contrast-enhanced destruction-replenishment US. Radiology. 2011;259: 406–413. 10.1148/radiol.10101339 21292869

[2] Lassau N, Koscielny S, Chami L, Chebil M, Benatsou B, Roche A, et al Advanced hepatocellular carcinoma: early evaluation of response to bevacizumab therapy at dynamic contrast-enhanced US with quantification—preliminary results. Radiology. 2011;258: 291–300. 10.1148/radiol.10091870 20980447

[3] Seitz K, Bernatik T, Strobel D, Blank W, Friedrich-Rust M, Strunk H, et al Contrast-enhanced ultrasound (CEUS) for the characterization of focal liver lesions in clinical practice (DEGUM Multicenter Trial): CEUS vs. MRI—a prospective comparison in 269 patients. Ultraschall Med. 2010;31: 492–499. 10.1055/s-0029-1245591 20652854

[4] Strobel D, Seitz K, Blank W, Schuler A, Dietrich CF, von Herbay A, et al Tumor-specific vascularization pattern of liver metastasis, hepatocellular carcinoma, hemangioma and focal nodular hyperplasia in the differential diagnosis of 1,349 liver lesions in contrast-enhanced ultrasound (CEUS). Ultraschall Med. 2009;30: 376–382. 10.1055/s-0028-1109672 19688669

[5] Strobel D, Seitz K, Blank W, Schuler A, Dietrich C, von Herbay A, et al Contrast-enhanced ultrasound for the characterization of focal liver lesions—diagnostic accuracy in clinical practice (DEGUM multicenter trial). Ultraschall Med. 2008;29: 499–505. 10.1055/s-2008-1027806 19241506

[6] Blomley MJ, Lim AK, Harvey CJ, Patel N, Eckersley RJ, Basilico R, et al Liver microbubble transit time compared with histology and Child-Pugh score in diffuse liver disease: a cross sectional study. Gut. 2003;52: 1188–1193. 12865280 10.1136/gut.52.8.1188 PMC1773750

[7] Albrecht T, Blomley MJ, Cosgrove DO, Taylor-Robinson SD, Jayaram V, Eckersley R, et al Non-invasive diagnosis of hepatic cirrhosis by transit-time analysis of an ultrasound contrast agent. Lancet. 1999;353: 1579–1583. 10334257 10.1016/S0140-6736(98)06373-9

[8] Kim MY, Suk KT, Baik SK, Kim HA, Kim YJ, Cha SH, et al. Hepatic vein arrival time as assessed by contrast-enhanced ultrasonography is useful for the assessment of portal hypertension in compensated cirrhosis. Hepatology. 2012. 10.1002/hep.25752 22473911

[9] Zhang CX, Hu J, Hu KW, Zhang C, Wang L, Xu JM. Noninvasive analysis of portal pressure by contrast-enhanced sonography in patients with cirrhosis. J Ultrasound Med. 2011;30: 205–211. 21266558 10.7863/jum.2011.30.2.205

[10] Tang A, Kim TK, Heathcote J, Guindi M, Jang HJ, Karshafian R, et al Does hepatic vein transit time performed with contrast-enhanced ultrasound predict the severity of hepatic fibrosis? Ultrasound Med Biol. 2011;37: 1963–1969. 10.1016/j.ultrasmedbio.2011.09.010 22033130

[11] Bendtsen F, Henriksen JH. Transit-time analysis in hepatic cirrhosis. Lancet. 1999;354: 598–599. 10470729 10.1016/S0140-6736(05)77953-8

[12] Li N, Ding H, Fan P, Lin X, Xu C, Wang W, et al Intrahepatic transit time predicts liver fibrosis in patients with chronic hepatitis B: quantitative assessment with contrast-enhanced ultrasonography. Ultrasound Med Biol. 2010;36: 1066–1075. 10.1016/j.ultrasmedbio.2010.04.012 20620694

[13] Lim AK, Taylor-Robinson SD, Patel N, Eckersley RJ, Goldin RD, Hamilton G, et al Hepatic vein transit times using a microbubble agent can predict disease severity non-invasively in patients with hepatitis C. Gut. 2005;54: 128–133. 15591518 10.1136/gut.2003.030965 PMC1774345

[14] Ridolfi F, Abbattista T, Busilacchi P, Brunelli E. Contrast-enhanced ultrasound evaluation of hepatic microvascular changes in liver diseases. World J Gastroenterol. 2012;18: 5225–5230. 10.3748/wjg.v18.i37.5225 23066317 PMC3468855

[15] Orlacchio A, Bolacchi F, Petrella MC, Pastorelli D, Bazzocchi G, Angelico M, et al Liver contrast enhanced ultrasound perfusion imaging in the evaluation of chronic hepatitis C fibrosis: preliminary results. Ultrasound Med Biol. 2011;37: 1–6. 10.1016/j.ultrasmedbio.2010.10.012 21144954

[16] Zhang L, Yin J, Duan Y, Yang Y, Yuan L, Cao T. Assessment of intrahepatic blood flow by Doppler ultrasonography: relationship between the hepatic vein, portal vein, hepatic artery and portal pressure measured intraoperatively in patients with portal hypertension. BMC Gastroenterol. 2011;11: 84 10.1186/1471-230X-11-84 21767412 PMC3156747

[17] Kim SY, Jeong WK, Kim Y, Heo JN, Kim MY, Kim TY, et al Changing waveform during respiration on hepatic vein Doppler sonography of severe portal hypertension: comparison with the damping index. J Ultrasound Med. 2011;30: 455–462. 21460144 10.7863/jum.2011.30.4.455

[18] Kim MY, Baik SK, Park DH, Lim DW, Kim JW, Kim HS, et al Damping index of Doppler hepatic vein waveform to assess the severity of portal hypertension and response to propranolol in liver cirrhosis: a prospective nonrandomized study. Liver Int. 2007;27: 1103–1110. 17845539 10.1111/j.1478-3231.2007.01526.x

[19] Baik SK, Kim JW, Kim HS, Kwon SO, Kim YJ, Park JW, et al Recent variceal bleeding: Doppler US hepatic vein waveform in assessment of severity of portal hypertension and vasoactive drug response. Radiology. 2006;240: 574–580. 16864678 10.1148/radiol.2402051142

[20] D'Amico G, Garcia-Pagan JC, Luca A, Bosch J. Hepatic vein pressure gradient reduction and prevention of variceal bleeding in cirrhosis: a systematic review. Gastroenterology. 2006;131: 1611–1624. 17101332 10.1053/j.gastro.2006.09.013

[21] European Association for the Study of the L. EASL clinical practice guidelines on the management of ascites, spontaneous bacterial peritonitis, and hepatorenal syndrome in cirrhosis. J Hepatol. 2010;53: 397–417. 10.1016/j.jhep.2010.05.004 20633946

[22] Sugimoto H, Kaneko T, Hirota M, Tezel E, Nakao A. Earlier hepatic vein transit-time measured by contrast ultrasonography reflects intrahepatic hemodynamic changes accompanying cirrhosis. J Hepatol. 2002;37: 578–583. 12399222 10.1016/s0168-8278(02)00264-7

[23] Rodriguez-Roisin R, Krowka MJ. Hepatopulmonary syndrome—a liver-induced lung vascular disorder. N Engl J Med. 2008;358: 2378–2387. 10.1056/NEJMra0707185 18509123

[24] Schenk P, Fuhrmann V, Madl C, Funk G, Lehr S, Kandel O, et al Hepatopulmonary syndrome: prevalence and predictive value of various cut offs for arterial oxygenation and their clinical consequences. Gut. 2002;51: 853–859. 12427789 10.1136/gut.51.6.853 PMC1773478

[25] Kim MY, Baik SK, Lee SS. Hemodynamic alterations in cirrhosis and portal hypertension. Korean J Hepatol. 2010;16: 347–352. 10.3350/kjhep.2010.16.4.347 21415576 PMC3304610

[26] Lim AK, Patel N, Eckersley RJ, Kuo YT, Goldin RD, Thomas HC, et al Can Doppler sonography grade the severity of hepatitis C-related liver disease? AJR Am J Roentgenol. 2005;184: 1848–1853. 15908541 10.2214/ajr.184.6.01841848

[27] Staub F, Tournoux-Facon C, Roumy J, Chaigneau C, Morichaut-Beauchant M, Levillain P, et al Liver fibrosis staging with contrast-enhanced ultrasonography: prospective multicenter study compared with METAVIR scoring. Eur Radiol. 2009;19: 1991–1997. 10.1007/s00330-009-1313-x 19259683

[28] Rockey DC. Hepatic blood flow regulation by stellate cells in normal and injured liver. Semin Liver Dis. 2001;21: 337–349. 11586464 10.1055/s-2001-17551

[29] Berzigotti A, Piscaglia F, Education E, Professional Standards C. Ultrasound in portal hypertension—part 2—and EFSUMB recommendations for the performance and reporting of ultrasound examinations in portal hypertension. Ultraschall Med. 2012;33: 8–32; quiz 30–31. 10.1055/s-0031-1299145 22322479

[30] Schneider AW, Kalk JF, Klein CP. Hepatic arterial pulsatility index in cirrhosis: correlation with portal pressure. J Hepatol. 1999;30: 876–881. 10365815 10.1016/s0168-8278(99)80142-1

[31] Taourel P, Blanc P, Dauzat M, Chabre M, Pradel J, Gallix B, et al Doppler study of mesenteric, hepatic, and portal circulation in alcoholic cirrhosis: relationship between quantitative Doppler measurements and the severity of portal hypertension and hepatic failure. Hepatology. 1998;28: 932–936. 9755228 10.1002/hep.510280406

